# Optimization of hippocampus sparing during whole brain radiation therapy with simultaneous integrated boost—tutorial and efficacy of complete directional hippocampal blocking

**DOI:** 10.1007/s00066-022-01916-3

**Published:** 2022-03-31

**Authors:** Ilinca Popp, Anca Ligia Grosu, Jamina Tara Fennell, Melissa Fischer, Dimos Baltas, Rolf Wiehle

**Affiliations:** 1 Department of Radiation Oncology, Medical Center, Faculty of Medicine, University of Freiburg, Robert-Koch Str. 3, 79106 Freiburg, Germany; 2 German Cancer Consortium (DKTK), Partner Site Freiburg, Freiburg, Germany

**Keywords:** WBRT, Radiotherapy planning, Brain metastases, Cognitive function, Toxicity

## Abstract

**Purpose:**

Hippocampus-avoidance whole brain radiotherapy with simultaneous integrated boost (HA-WBRT+SIB) is a complex treatment option for patients with multiple brain metastases, aiming to prevent neurocognitive decline and simultaneously increase tumor control. Achieving efficient hippocampal dose reduction in this context can be challenging. The aim of the current study is to present and analyze the efficacy of* complete directional hippocampal blocking* in reducing the hippocampal dose during HA-WBRT+SIB.

**Methods:**

A total of 30 patients with multiple metastases having undergone HA-WBRT+SIB were identified. The prescribed dose was 30 Gy in 12 fractions to the whole brain, with 98% of the hippocampus receiving ≤ 9 Gy and 2% ≤ 17 Gy and with SIB to metastases/resection cavities of 36–51 Gy in 12 fractions. Alternative treatment plans were calculated using complete directional hippocampal blocking and compared to conventional plans regarding target coverage, homogeneity, conformity, dose to hippocampi and organs at risk.

**Results:**

All alternative plans reached prescription doses. Hippocampal blocking enabled more successful sparing of the hippocampus, with a mean dose of 8.79 ± 0.99 Gy compared to 10.07 ± 0.96 Gy in 12 fractions with the conventional method (*p* < 0.0001). The mean dose to the whole brain (excluding metastases and hippocampal avoidance region) was 30.52 ± 0.80 Gy with conventional planning and 30.28 ± 0.11 Gy with hippocampal blocking (*p* = 0.11). Target coverage, conformity and homogeneity indices for whole brain and metastases, as well as doses to organs at risk were similar between planning methods (*p* > 0.003).

**Conclusion:**

Complete directional hippocampal blocking is an efficient method for achieving improved hippocampal sparing during HA-WBRT+SIB.

## Introduction

Radiation therapy is an essential treatment pillar for patients with multiple brain metastases [1]. However, especially in the case of the classical whole brain radiation therapy (WBRT), this treatment can also lead to substantial deterioration in cognitive function and quality of life, which is often permanent and noticeable very early after treatment [2–4].

The irradiation of the hippocampus and the hippocampal neural stem cell niche has been considered the most important cause of neurocognitive decline after cerebral radiotherapy [5, 6]. In line with these preclinical findings, clinical trials have demonstrated that hippocampal avoidance during WBRT (HA-WBRT) can achieve a significant reduction in cognitive failure rates, leading to less deterioration in executive function, learning and memory [7–9]. Furthermore, maximal dose de-escalation to the hippocampi was also proven feasible by omitting WBRT altogether and exclusively performing radiosurgeries to the metastases [10].

On the other hand, poorly controlled intracranial tumor and high cerebral metastatic burden are known to have a major negative impact on cognitive functions [11, 12]. Conventional WBRT with a standard dose of 30 Gy in 10 fractions substantially reduces neurological death rates [13], but ensures only a modest local tumor control of existing metastases, with a median time to local brain failure of 6 months [1]. While multiple radiosurgeries lead to an excellent local tumor control (over 80%), the rate of new lesions was shown to be over 60% in the first year [10]. Therefore, a combination of WBRT and simultaneous or subsequent dose escalation to the metastases can significantly reduce both local and distant intracranial disease progression [14, 15].

Aiming to prevent neurocognitive decline both through dose restriction to the hippocampi and through an increased intracranial tumor control, the complex treatment option of WBRT with hippocampal sparing and simultaneous integrated boost to the metastases (HA-WBRT+SIB) was developed [16]. The combination is being evaluated in the ongoing prospective randomized HIPPORAD trial (NOA-14, ARO 2015‑3, DRKS00004598). A total dose of 30 Gy in 12 fractions is being applied to the whole brain, a SIB of 51 Gy in 12 fractions to the metastases, while maintaining the dose to 98% of the hippocampal volume (D98%) below 9 Gy and the dose to 2% of the hippocampal volume (D2%) below 17 Gy [17]. Therefore, especially in the case of SIB for metastases located in the vicinity of the hippocampi, significant modulation and highly conformal radiotherapy is required.

Maximal dose reduction to the hippocampi has been proven essential for cognitive protection. Preclinical data have suggested that every additional gray applied to the hippocampus can have a significant negative impact [5]. Furthermore, a longitudinal volumetric analysis comparing hippocampal atrophy following HA-WBRT as opposed to conventional WBRT demonstrated significant atrophy after both treatment modalities [18]. The volume decline seen after HA-WBRT was significantly lower than after WBRT, but, with an estimated annual rate of 1.6%, still higher than what would be expected for the included population. In the phase III clinical trial of Brown et al., there was a considerable proportion of over 50% of patients, who suffered measurable cognitive decline despite hippocampal avoidance [9]. Therefore, achieving a steeper dose gradient for hippocampal sparing in WBRT and WBRT+SIB appears essential for the preservation of cognitive functions and quality of life.

For hippocampal sparing during WBRT and WBRT+SIB, a hippocampus avoidance region (HAR) is defined as the hippocampus plus 5–7 mm [7, 9, 16, 17, 19] and assigned higher priorities relative to other objectives in radiation treatment planning. This often leads to an inhomogeneity in dose distribution for the target volume and sometimes fails to achieve proper hippocampal avoidance. Complete blocking of radiation beams during dose optimization is feasible in radiation treatment planning, but is currently used only for a restricted number of indications—such as avoiding dose to pacemakers and hip endoprostheses. To our knowledge, there are currently no guidelines or systematic evaluations for the use of hippocampal blocking for reduction of the hippocampal exposure during WBRT or WBRT+SIB.

The aim of the current study was therefore to present and investigate the feasibility of complete hippocampal blocking in the radiation treatment planning of HA-WBRT+SIB and compare the resulting parameters with conventional planning methods. Our goal was to determine the advantages and limitations of this method in meeting the defined treatment planning objectives using volumetric modulated arc therapy (VMAT) for patients with multiple brain metastases.

## Materials and methods

### Patient sample

The current study was approved by the local ethics committee. We identified 30 patients with multiple metastases of solid tumors, having received conventionally planned HA-WBRT+SIB in the Department of Radiation Oncology of the Medical Center—University of Freiburg. Patients had on average 5 lesions amenable to SIB (metastases ≥ 5 mm and resection cavities), with no lesions within the hippocampus. Detailed characteristics of the selected patients are listed in Table 1. In 28 patients, lesions were located at least 7 mm away from the hippocampus (median 19.15 mm, range 7.3–40 mm). Metastases were in close proximity to the hippocampus in 2 patients (minimal distance 0 mm and 4 mm).

**Table 1:** Clinical details of selected patients

Patient characteristics	
Age (years), median, range	57.5, 39–81
Gender (*n*), f/m	19/11
*Primary tumor (n)*	
Malignant melanoma	11
Lung cancer	9
Breast cancer	8
Gastrointenstinal cancer	2
*Metastases/resection cavities (n)* median, range	5 (3–11)
*PTV of metastases/resection cavities (ml)* median, range	6.3 (1–138.2)
*PTV of whole brain (ml)* median, range	1701.2 (1276.7–2166.5)
*Total hippocampal volume (ml)* median, range	4.3 (2.8–6.6)

*f/m* female/male, *PTV* planning target volume

### HA-WBRT+SIB radiation treatment planning

The goals of the radiation treatment planning were (i) homogeneous whole brain dose distribution, (ii) maximal hippocampal sparing, (iii) highly conformal dose escalation to brain metastases and (iv) protection of predefined organs at risk (brainstem, optical chiasm and optical nerves, eyes, inner ears and lenses).

For radiation treatment planning, patients underwent computed tomography (CT) in thermoplastic mask immobilization (BrainLab, Feldkirchen, Germany). Contrast-enhanced T1-weighted magnetic resonance imaging scans for treatment planning [20] and CT images were rigidly coregistered based on mutual information in the contouring system (iPlan RT Image 4.1.1, BrainLab, Feldkirchen, Germany) and served for target volume and organ at risk delineation. Segmented volumes were then transferred to the Eclipse planning system (Varian Medical Systems, Inc., version 15.6, Palo Alto, CA, USA).

The planning target volume (PTV) for the brain (PTV_WB_) was defined as the whole brain (clinical target volume [CTV]) plus 3 mm, excluding PTVs of metastases and the HAR. The prescribed dose for the brain PTV was 30 Gy in 12 fractions on 95% of the volume. The PTV amenable for SIB (PTV_BM_) was defined as the gross tumor volume (GTV) of brain metastases and the CTV of resection cavities with a 1 mm and 2 mm isotropic margin, respectively, and was treated with a simultaneous integrated boost (SIB) of 51 Gy/42 Gy/36 Gy in 12 fractions on 95% of the volume, depending on size and location.

The hippocampi were delineated according to the RTOG 0933 contouring atlas [7]. The HAR was defined as a 7 mm three-dimensional margin around the hippocampus, as this was previously identified as the optimal margin for hippocampal sparing in HA-WBRT+SIB [16]. The best possible hippocampal avoidance was attempted in all cases. If metastases were located outside the HAR, the aim was to comply with the hippocampus constraints of the currently ongoing HIPPORAD trial: D98% ≤ 9 Gy, D2% ≤ 17 Gy [17]. If metastases were located within the HAR, the best possible hippocampal protection was strived for, while maintaining appropriate coverage of PTV_BM_. Eyes, lenses, optic nerves, optic chiasm, inner ears and brainstem were additionally defined as organs at risk. Constraints were analogous to those in the HIPPORAD trial: for brainstem, inner ears, eyes, optical nerves and chiasma D2% ≤ 33 Gy in 12 fractions, whereas for lenses the constraint was D2% ≤ 7 Gy in 12 fractions [17].

For all treatment plans the prescription dose of 30 Gy (100%) was set to cover 95% of the PTV_WB_. The initial, conventional plans were optimized in Eclipse version 10.0 for 6 MV photons for the Varian linear accelerator with a Millenium 120-leaf multileaf collimator (Varian Medical Solutions). The VMAT planning was based on 2–4 coplanar whole arcs in clockwise and counterclockwise directions. The collimator angle was individually chosen between 30° and 45°, as described previously [16]. An initial set of constraints was defined to include target coverage and sparing of organs at risk. The main goal was to reduce the dose to the hippocampus without compromising the coverage of PTV_WB_ and PTV_BM_. Conventional hippocampal avoidance was performed by assigning higher priority to the HAR relative to other objectives and by creating additional structures for the optimization.

**Fig. 1:**
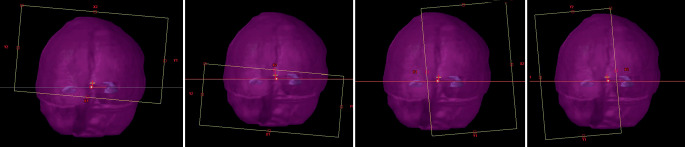
Example of treatment fields for hippocampus-avoidance whole brain radiotherapy with simultaneous integrated boost (HA-WBRT+SIB). Two complete arcs with a collimator angle near 0° (e.g., 5°) and two complete arcs with a collimator angle near 90° (e.g., 85°) are defined and the field openings are adapted to the PTV_WB_. Afterwards the x‑jaws are closed such that two arcs with identical collimator angle only have a small overlap (2–3 cm). *Light purple* whole brain, *dark purple* hippocampi, *PTV*_*WB*_ planning target volume of the whole brain

The treatment plans which used the complete blocking of the hippocampi were optimized in Eclipse version 15.6. Starting with version 15, the optimization algorithm allows blocking radiation from structures, either before the beam reaches one of the target volumes (entry or directional blocking) or completely (entry + exit or complete blocking). Four complete coplanar arcs with collimator angles of 5° (2 arcs) and 85° (2 arcs) were used. The primary x‑jaws were set such that the field openings of the two arcs with identical collimator angle result in a small overlap around the isocenter, which is placed between the two hippocampi (Fig. 1). Thereby the maximum field opening in the x‑direction is below 12 cm, which increases the degrees of freedom for fluency modulation. Furthermore, the leaves can easily block the hippocampi without blocking large regions of the PTV_WB_. A template for the optimization objectives was used as a starting point. The objectives were then further adjusted during the optimization process according to the evolution of the dose distribution. Step-by-step instructions for hippocampal blocking are presented in Table 2.

**Table 2:** Instructions for the use of hippocampal blocking in the radiation treatment planning of hippocampus-avoidance whole brain radiation therapy with simultaneous integrated boost (HA-WBRT+SIB)

**1**	**Define structures for optimization**					
	**HAR**: the union of the two hippocampi with an isotropic margin of 7 mm					
	**Ring_1** **cm**: a 1 cm isotropic expansion of HAR from which HAR is subtracted					
	**BMs**: the union of all PTV_BM_ amenable to SIB					
	**BMs +5** **mm**: a 5 mm isotropic expansion of BMs					
	**PTV**_**WB**_**-HAR**: PTV_WB_ with HAR subtracted					
	**PTV**_**WB**_**-HAR-BMs**: PTV_WB_ with HAR and BMs +5 mm subtracted					
**2**	**Set parameters for treatment fields**					
	The isocenter is placed between the two hippocampi					
	Two complete arcs with a collimator angle near 0° (e.g., 5°) plus two complete arcs with a collimator angle near 90° (e.g., 85°)					
	In the first step the field openings are adapted to the PTV_WB_. In the second step the x‑jaws are closed such that the two arcs with identical collimator angle only have a small overlap between 2 cm and 3 cm (Fig. 1). This allows the MLC to block the hippocampi at all angles without also blocking larger parts of the PTV					
**3**	**Set objectives—**e.g., according to the following template: (prescription dose for PTV_WB_/PTV_BM_1,2,3_ _…_/PTV_BM_4,5,6_ _…_ of 30 Gy/51 Gy/42 Gy)					
	*Structure*	*Type*	*Dose [Gy]*	*Priority*	*gEUD*_*a*_	*Blocking*
	PTV_WB_-HAR-BMs	Upper	40.00	1000	–	No
		Upper	30.00	1000	–	
		Lower	27.00	1000	–	
		Lower	29.20	1000	–	
		Lower	29.50	1000	–	
		Lower	28.50	1000	–	
		Upper gEUD	33.00	1000	40.0	
		Upper gEUD	32.00	1000	20.0	
		Upper gEUD	31.00	1000	10.0	
	GTV_BM_1,2,3_ _…_	Lower	51.00	1000	–	No
	PTV_BM_1,2,3_ _…_	Upper	53.00	1000	–	No
		Lower	51.00	1000	–	
		Lower	50.00	1000	–	
		Upper gEUD	52.00	1000	40.0	
		Lower gEUD	51.50	1000	−40.0	
	GTV_BM_4,5,6_ _…_	Lower	43.00	1000	–	No
	PTV_BM_4,5,6_ _…_	Upper	43.00	1000	–	No
		Lower	41.80	1000	–	
		Lower	41.30	1000	–	
		Upper gEUD	43.00	1000	40.0	
		Lower gEUD	42.50	1000	−40.0	
	Hippocampus left, right	Upper	8.00	800	–	**Yes**
		Upper	6.00	800	–	
		Mean	8.00	700	–	
	Brainstem	Upper	33.00	1000	–	No
		Lower	28.50	1000	–	
		Upper gEUD	30.30	1000	40.0	
		Upper gEUD	30.00	600	40.0	
	Chiasm	Upper	31.00	1000	–	No
		Lower	28.01	100	–	
		Upper gEUD	30.50	1000	40.0	
	Optic nerves left, right	Upper	31.00	1000	–	No
	Eye left, right	Mean	7.00	600	–	No
		Upper gEUD	11.00	600	10.0	
	Lens left, right	Upper	5.00	800	–	No
		Upper gEUD	5.00	1000	40.0	
		Upper gEUD	4.00	800	5.0	
	Ring_1 cm	Upper	33.00	700	–	No
		Lower	27.50	700	–	
		Lower	28.50	700	–	
		Lower	29.00	900	–	
		Lower	28.50	900	–	
		Upper gEUD	33.00	600	40.0	
	z_low (see below)	Lower	29.00	1000	–	No
		Lower	28.50	1000	–	
		Lower	28.00	1000	–	
	z_high (see below)	Upper	33.00	1000	–	No
		Upper gEUD	32.00	1000	40.0	
	z_out (see below)	Upper gEUD	26.00	800	10.0	No
		Upper gEUD	29.00	1000	40.0	
**4**	**Fine tuning**					
	Convert 28.5 Gy isodose to structure and subtract the result from PTV_WB_-HAR to define the areas in which the PTV is underdosed. The corresponding volume is found as **z_low** in the objective template					
	Convert 28.5 Gy isodose to structure and crop the result from PTV_WB_ to define the areas outside the PTV which receive a high dose. The corresponding volume is found as **z‑out** in the objective template					
	Convert 32 Gy isodose to structure and crop the result from BMs +5 mm with an additional margin of 5 mm to define the areas in which the PTV is overdosed. The corresponding volume is found as **z‑high** in the objective template					
	Optimize and repeat these steps until the result is satisfactory. Similar volumes can of course be used to optimize the dose inside the SIB volumes. However, according to our experience this is hardly necessary					

*WB* whole brain, *SIB* simultaneous integrated boost, *HAR* hippocampus avoidance region, *BMs* brain metastases, *RES* resection cavity, *PTV* planning target volume, *GTV* gross tumor volume, *MLC* multileaf collimator, *gEUD* generalized equivalent uniform dose, *gEUD*_*a*_ weighting parameter *a* of the generalized equivalent uniform dose

### Treatment planning evaluation

Treatment plans were evaluated on target coverage, homogeneity, conformity and dose distribution to the hippocampus and organs at risk. One of the limitations of dose escalation for multiple brain metastases is the mean dose to the whole brain. Therefore, the mean dose received by the PTV_WB_ was also included in the evaluation, with 35 Gy considered the dose tolerance maximum for a fractionation of 2.5 Gy.

Target coverage (TC) for PTV_WB_ and PTV_BM_ was determined by the percentage of the volume of the target receiving more than 95% of the prescribed dose. For perfect coverage, TC equals 1.0. Homogeneity index was defined according to ICRU83 as the D2% minus D98% divided by the median dose to the target volume (D50%) [21]: the smaller the homogeneity index, the more homogeneous the underlying dose distribution. The RTOG conformity index was used for PTV_WB_ and PTV_BM_ and defined as the reference isodose volume divided by the target volume [22]. A conformity index between 1 and 2 was considered appropriate.

Sparing of the hippocampus was evaluated considering the mean dose, the D98% and D2%, as well as the equivalent dose in 2 Gy fractions (EQD2) with an α/β = 2 Gy for 40% of both hippocampi (D40%). Protection of other normal tissue was quantified using the D2%.

Statistical analysis was performed in SPSS software (version 27.0; IBM, Armonk, NY, USA) using paired t tests and Bonferroni correction and a significance level at *p* < 0.003.

## Results

All plans reached prescription doses. PTV_WB_ was on average 1701.2 ml (range 1276.70–2166.50 ml) and had similar average TC values for both methods: 92.85 ± 3.92% for conventional planning and 93.96 ± 5.53% for hippocampal blocking (*p* = 0.39). Homogeneity indices were also comparable, with values of 0.27 ± 0.11 and 0.27 ± 0.21, respectively (*p* = 0.95). Conventional planning and hippocampal blocking led to similar conformity indices of 1.06 ± 0.09 and 1.07 ± 0.06, respectively (*p* = 0.19). The mean dose to PTV_WB_ was kept under 35 Gy for all patients. The average mean dose was 30.52 ± 0.80 Gy with conventional planning and 30.28 ± 0.11 Gy with hippocampal blocking (*p* = 0.11).

Of 30 patients, the prescription dose to the metastatic lesions was 51 Gy in 18 patients, 51 Gy and 42 Gy in 10 patients, 42 Gy for 1 patient and 36 Gy for 1 patient. The average PTV_BM_ was 21.55 ml (range 1.00–138.20 ml). The PTV coverage for PTV_BM_ was excellent for both planning methods, with a mean TC value of 98.26 ± 2.38% for conventional planning and 99.21 ± 1.30% for hippocampal blocking (*p* = 0.05). The homogeneity index was also equally good, with values of 0.10 ± 0.02 and 0.09 ± 0.02 respectively (*p* = 0.004). The conformity indices were 1.51 ± 0.47 with conventional planning and 1.53 ± 0.36 with hippocampal blocking (*p* = 0.89).

**Fig. 2:**
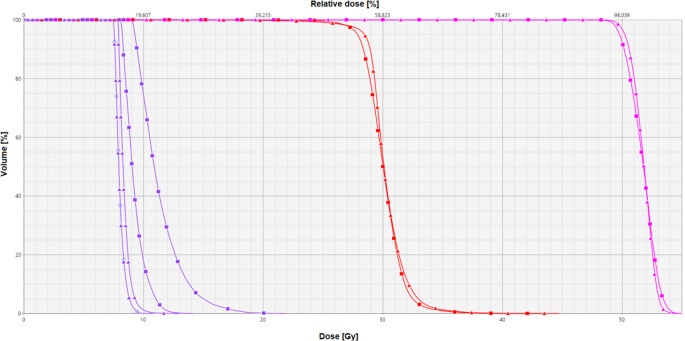
Comparative dose volume–histogram (DVH) displaying the outcome of conventional planning (■) and hippocampal blocking (▲) for hippocampus-avoidance whole brain radiation therapy with simultaneous integrated boost (HA-WBRT+SIB). *Purple* Hippocampi (left and right), *red* PTV_WB_ receiving 30 Gy, *pink* PTV_BM_ receiving 51 Gy

**Fig. 3:**
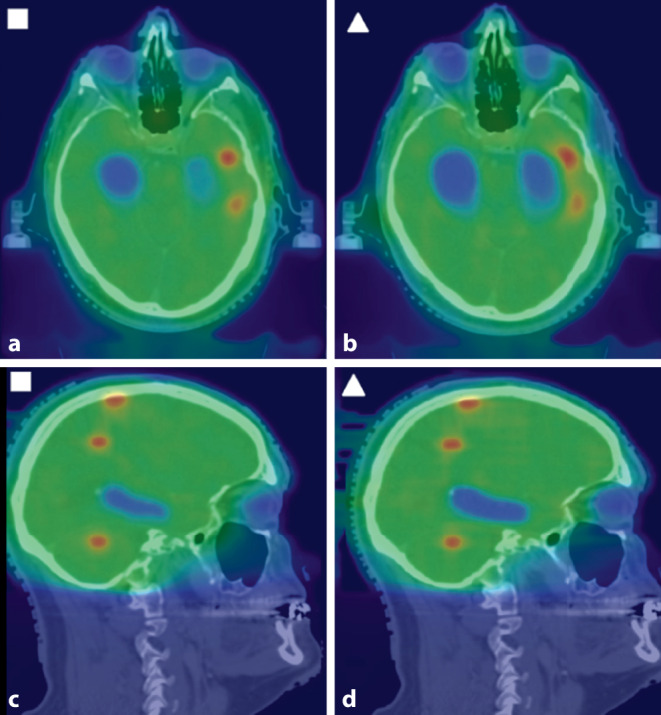
Dose distribution on axial (**a**, **b**) and sagittal (**c**, **d**) planning computed tomography (CT) images, displaying the outcome of conventional planning (■) and of the hippocampal blocking method (▲). Especially the left hippocampus shows improved protection in spite of the vicinity to metastases with dose escalation

The average volume of the hippocampi was 4.44 ml (2.80–6.6 ml); 80% of conventional plans and 97% of hippocampal blocking plans adhered to hippocampal constraints. The hippocampal blocking method achieved a much steeper and homogeneous dose reduction in both hippocampi, as can be seen in the example dose–volume histogram (DVH) in Fig. 2. The corresponding spatial dose distribution is presented in Fig. 3. The mean dose to the hippocampus was 10.07 ± 0.96 Gy with conventional planning and 8.79 ± 0.99 Gy with hippocampal blocking (*p* < 0.0001). D98% was on average 8.49 ± 0.50 Gy with conventional planning and 7.87 ± 0.28 Gy with hippocampal blocking (*p* < 0.0001), while D2% was 14.48 ± 3.38 Gy and 10.88 ± 3.48 Gy respectively (*p* < 0.0001). All in all, the mean hippocampal dose could be reduced by 1.28 ± 0.64 Gy through hippocampal blocking. This allowed adherence to set constraints in all but one patient, the only one with a metastasis immediately adjacent to the hippocampus. In this case, a mean dose of 9.8 Gy EQD2 α/β = 2 was achieved.

Constraints for organs at risk were respected with both planning methods, with no dose increase brought on by hippocampal blocking (Table 3).

**Table 3:** Dose to organs at risk. For paired structures, the highest value is listed

Organs at risk	Conventional planning		Hippocampal blocking		Statistical difference (paired t‑test)
	Dose in 12 fractions	EQD2 α/β = 2	Dose in 12 fractions	EQD2 α/β = 2	
*Hippocampus*					
Dmean ± SD (Gy)	10.07 ± 0.96	7.14 ± 0.49	8.79 ± 0.99	6.00 ± 0.52	*p* < 0.00001
D2% ± SD (Gy)	14.48 ± 0.38	11.61 ± 0.93	10.88 ± 0.48	7.91 ± 1.99	*p* < 0.00001
D98% ± SD (Gy)	8.49 ± 0.50	5.75 ± 0.26	7.87 ± 0.28	5.23 ± 0.14	*p* < 0.00001
D40% ± SD (Gy)	9.95 ± 0.76	7.04 ± 0.39	8.58 ± 0.63	5.82 ± 0.32	*p* < 0.00001
*Brainstem*	32.81 ± 0.95	38.83 ± 0.49	31.87 ± 1.03	37.10 ± 0.54	
D2% ± SD (Gy)					*p* = 0.0014
*Optical nerves*	32.30 ± 2.14	37.89 ± 1.17	31.91 ± 2.48	37.17 ± 1.37	
D2% ± SD (Gy)					*p* = 0.122
*Chiasm*	31.66 ± 4.12	36.71 ± 2.41	30.72 ± 4.20	35.02 ± 2.47	
D2% ± SD (Gy)					*p* < 0.00001
*Eyes*	20.47 ± 5.05	18.96 ± 2.84	17.25 ± 3.83	14.82 ± 2.22	
D2% ± SD (Gy)					*p* = 0.0013
*Inner ears*	31.68 ± 1.30	36.75 ± 0.69	31.81 ± 1.62	36.99 ± 0.86	
D2% ± SD (Gy)					*p* = 0.611
*Lenses*	6.09 ± 0.58	3.82 ± 0.30	6.22 ± 0.89	3.92 ± 0.46	
D2% ± SD (Gy)					*p* = 0.436

*EQD2 α/β* *=* *2* equivalent dose in 2 Gy fractions, considering an α/β ratio of 2, *Dmean* mean dose; D2%, near-maximum dose, *D98%* near-minimum dose, *D40%* dose applied to 40% of the bilateral hippocampus

## Discussion

To our knowledge, this is the first study to use complete directional hippocampal blocking for hippocampal avoidance during HA-WBRT+SIB. We could demonstrate that this radiation treatment planning method achieves improved hippocampal sparing in an efficient manner compared to conventional optimization strategies. The use of this method led to a steeper dose reduction, while allowing successful simultaneous dose escalations on multiple brain metastases.

The hippocampus is a central element in the formation of memory, managing to process an enormous amount of information through extraordinary synaptic plasticity [23]. Radiation therapy to the hippocampus was shown to lead to dose-dependent atrophy [18, 24] and dose-dependent cognitive deficits [8, 25]. Among all cortical regions, the areas responsible for cognitive functions, and in particular the hippocampus, have the highest sensibility and experience the most significant radiation-induced atrophy [26]. Maximal protection of the hippocampus is thus imperative. Furthermore, this also appears to be safe, taking into consideration the low risk of developing hippocampal metastases of below 5% [27, 28] and the rather unproblematic salvage options.

Accordingly, various hippocampal constraints have been deemed important for preserving cognitive functions in cerebral irradiation. A dosimetric analysis of stereotactic fractionated radiotherapy for low-grade adult brain tumors revealed an equivalent dose of 7.3 Gy in 40% of the bilateral hippocampi (D40%, EQD2 α/ß = 2) as cut-off for the occurrence of long-term cognitive impairment [8]. In the current study, hippocampal blocking managed to significantly reduce the hippocampal dose beyond this cut-off, suggesting that it may result in more efficient cognitive protection. The RTOG 0933 and the NRG Oncology CC001 trials imposed 100% of the hippocampus did not exceed a dose of 9 Gy (6.5 Gy EQD2 α/ß = 2), with a maximal dose lower than 16 Gy in 10 fractions (14.4 Gy EQD2 α/ß = 2) [7, 9]. In a trial for patients with small-cell lung cancer and prophylactic cranial irradiation, constraints for the hippocampal mean dose were set even lower, to 8 Gy in 10 fractions (5.6 Gy EQD2 α/ß = 2) [29].

In the current work, constraints were analogous to the HIPPORAD trial for HA-WBRT+SIB [17]. Hippocampal blocking managed to significantly reduce hippocampal dose, resulting in the adherence to set constraints in 5 additional patients compared to conventional planning. The method explored here managed to produce a steeper dose gradient in all patients, including those with metastases located in the vicinity of the hippocampi. In spite of the strong dose modulation, the TC, conformity and homogeneity indices for whole brain and metastases obtained in this trial were adequate and comparable with the results of previous studies [16, 30–32].

To our best knowledge, blocking radiation beams has not been routinely used for cerebral radiation treatment planning. In recent years, complete blocks have been explored for better sparing of the heart and lungs in the irradiation of breast, lung and esophageal cancer [33–35]. Similar to our results, this method not only achieved similar PTV coverage, homogeneity and dose conformity but also allowed better protection of organs at risk. A limitation of this approach may only entail an initially longer planning time. Therefore, in order to facilitate the broader usage of this method, we provided step-by-step recommendations for using hippocampal blocking during HA-WBRT+SIB treatment planning.

An important limitation of this work is that the clinical benefit of the achieved hippocampal dose reduction was not analyzed. Whether a higher degree of hippocampal sparing will have a significant impact on neurocognitive functions remains to be evaluated in prospective studies, such as the ongoing HIPPORAD trial. Furthermore, although the hippocampus is crucial for memory consolidation, it is not the only structure of interest. Other brain substructures, including the corpus callosum and frontal white matter, play a key role in specific neurocognitive functions, such as attention and processing speed [36, 37]. Even though hippocampal blocking did not significantly affect whole brain dose homogeneity and conformity, future research is warranted to evaluate possible dose variations in these particular structures and their clinical implications.

## Conclusion

The current study demonstrates that complete directional hippocampal blocking can effectively minimize hippocampal dose compared to conventional planning, while maintaining excellent target coverage, conformal dose escalation to metastases and acceptable whole brain dose homogeneity. These results could contribute to a significant change in standard radiation treatment planning, by a consistent implementation of hippocampal blocking for sparing hippocampus during HA-WBRT+SIB.
